# Genomic diversity and tracing of Paenibacillus larvae in Australia: implications for American foulbrood outbreak surveillance in low-diversity populations

**DOI:** 10.1099/mgen.0.001374

**Published:** 2025-05-06

**Authors:** Paul Worden, Ashlea Webster, Khushbu Gandhi, Risha Gupta, Ania T. Deutscher, Michael Hornitzky, Daniel Ross Bogema

**Affiliations:** 1 NSW Department of Primary Industries, Elizabeth Macarthur Agricultural Institute, Woodbridge Rd, Menangle, NSW, Australia; 2 Australian Centre for Genomic Epidemiological Microbiology, University of Technology Sydney, 17 Broadway, Ultimo, NSW, Australia

**Keywords:** American foulbrood, *Apis mellifera*, core genome, honeybee, microbial pathogens, multilocus sequence type, *Paenibacillus larvae*, phylogenetics, SNPs, whole-genome sequencing

## Abstract

*Paenibacillus larvae* is the causative agent of American foulbrood (AFB) in honeybees (*Apis mellifera*) and a devastating pathogen for honey and pollination industries worldwide. Despite this threat, a genomic survey of *P. larvae* has not been attempted within Australia. To examine the diversity of Australian populations, we sequenced 368 *P*. *larvae* genomes sourced primarily from south-eastern Australia. Multilocus sequencing typing analysis identified only 4 sequence types across all 368 samples, with 2 sequence types (ST18 and ST5) representing 96% of all isolates. In comparison to European-sourced *P. larvae*, sequences revealed much less genetic diversity in Australian isolates. However, Australian genotypes were very similar to those found in New Zealand populations. All Australian isolates were identified as enterobacterial repetitive intergenic consensus (ERIC) type I. To determine the feasibility of a genomic tracing system in a low-diversity genetic background, we investigated core-genome SNP (cgSNP) genotyping of isolates from a single beekeeper and from isolates across multiple apiaries and sample sites. We identified highly related cgSNP clusters, one with known epidemiological links, but another highly related cluster spanned several decades. Results strongly suggest that cgSNP analysis does have the discriminatory power to assist in the trace-forward and trace-back of AFB outbreaks, but importantly, the inclusion of background sequences and careful consideration of multiple analysis methods are required to avoid erroneous conclusions.

## Data Summary

The whole-genome sequencing reads and subsequent genome assemblies for the 368 bacterial isolates reported in the study have been deposited at DDBJ/ENA/GenBank under BioProject accession number PRJNA1117589. A list of SRA, isolate and enterobacterial repetitive intergenic consensus-type reference genome identifiers used in this study is included in Tables S1–S3 (available in the online Supplementary Material). Quality control data for isolate and SRA assemblies are in Table S4(A and B). *In silico* multilocus sequencing typing (MLST) results for assemblies from our isolates, NCBI reference genomes and SRA genomes downloaded in 2022 are in Table S5. MLST results for New Zealand isolates were taken from Binney *et al.* [1].

**Impact Statement**

American foulbrood (AFB) is a highly infectious and notifiable disease of the honeybee brood, caused by the bacteria *Paenibacillus larvae*. This article examines the genetic diversity found within Australian *P. larvae* populations in comparison with internationally sourced *P. larvae* genomes, especially Europe and New Zealand. The frequency of AFB introduction to Australia and whether existing genomic typing methods can improve trace-forward and trace-back of Australian AFB outbreaks was also considered. Our results indicate that AFB in Australia and New Zealand is both genetically similar but less diverse than European outbreaks. We found that genomic analysis has the discriminatory power to trace AFB outbreaks to a single strain, but care must be taken to accurately link positive detections, and closely related background sequences should be included in any analysis. These results also suggest that other highly discriminatory genomic typing methods could be employed in Australian AFB outbreak tracing, such as core-genome multilocus sequencing typing and multilevel genome typing.

## Introduction

American foulbrood (AFB) is a highly contagious brood disease of the honeybee (*Apis mellifera*) caused by the spore-forming, gram-positive bacterium *Paenibacillus larvae* [2,3]. Internationally, AFB is a decimator of bee colonies and represents a threat to the multi-billion-dollar Australian honey and wax industries. Internationally, AFB also threatens many agricultural crops whose pollination is either assisted by, or requires, the honeybee [4,5]. To reduce this impact, some countries use antibiotics to suppress AFB, whilst other jurisdictions, including all mainland states of Australia, have banned the use of antibiotics against AFB [6]. The spore-forming nature of *P. larvae* results in antibiotics only masking the disease and encouraging the evolution of antibiotic resistance [7,9]. Antibiotic residues in honey can also alter its properties and affect its commercial value [10]. Whilst the recent development of an oral vaccine for honeybees against *P. larvae* is promising [11], there is no current effective method of controlling AFB in Australia other than burning or gamma-irradiation of contaminated hive material and diseased colonies [12].

The 20–40 years of durability and longevity of *P. larvae* spores [13], common beekeeping practices and the global trade of bees and honey have facilitated the international spread of AFB. *P. larvae* can spread through natural means, such as drifting, robbing or beekeeping practices. Drifting is when bees accidentally return to the wrong hive [14], whilst robbing involves bees actively attacking and stealing resources from a weaker colony [15]. However, there is growing evidence that beekeeping practices, such as transporting and reuse of hive material and transferring bees between colonies, are the more common route of transmission [16]. AFB outbreaks are observed to occur more frequently where bee colony densities are higher than under natural conditions [17,18], although there is some disagreement [19].

Currently, the destruction of AFB-affected colonies is the only viable long-term control method [20,21]. As such, timely and effective trace-back and trace-forward of *P. larvae* remains critical to limit the scope and impact of outbreaks [22,23]. Enterobacterial repetitive intergenic consensus (ERIC) typing is one of the most common typing methods because it correlates well with distinct pathologies, yet to date, only five ERIC types have been discovered. ERIC-I is highly virulent to a honeybee colony [21,24], whilst ERIC-II infection is less virulent to a colony and sometimes survivable [3,21]. ERIC-III, ERIC-IV and the more recently discovered ERIC-V [25] appear to have limited association with honeybee pathogenesis [24,26]. In comparison with ERIC typing, the seven-gene *P. larvae* method of multilocus sequence typing (MLST) has greater genotyping discriminatory power. However, it is unable to resolve outbreaks down to the taxonomic level of strain [27] and so has limited value in tracing AFB outbreaks.

More recently, the reduced costs of whole-genome sequencing (WGS) technologies have allowed the development of new typing methods using several phylogenomic techniques [28]. This has resulted in both improved repeatability and greater discriminatory power in both observing the evolutionary relationships between the different strains of bacteria and determining outbreak clusters [29]. Techniques such as core-genome SNP (cgSNP) analysis, pangenome analysis and core-genome MLST (cgMLST) have all been used to help understand evolutionary relationships and identify bacterial strains [23,30,32]. To date, the most discriminative and sensitive WGS-based methods are cgSNP comparisons, which require an appropriate reference genome and that all WGS reads and assemblies have passed quality control (QC) analyses [33].

For the study reported here, we examined 368 *P*. *larvae* genomes primarily from south-eastern (SE) Australia, using several WGS-based phylogenomic approaches to characterize the genetic makeup of AFB outbreaks. Our Australian *P. larvae* genomes were sequenced primarily from AFB-positive isolates obtained from samples submitted to the Elizabeth Macarthur Agricultural Institute (EMAI) for AFB testing. Some of these samples were obtained as far back as the 1980s, but the majority were collected between 2018 and 2022.

## Methods

### Collection of *P. larvae* at EMAI

*P. larvae* isolates were obtained from a combination of historical isolates or current samples (mostly larval bee smear but some honey samples) that had been submitted to EMAI (the New South Wales state veterinary laboratory) to test for American foulbrood. Fourteen of these isolates were from cultures originating from internationally sourced AFB samples.

### Culture of *P. larvae*

Historical freeze-dried isolates were collected by cotton dry swabs and streaked onto blood agar plates produced in-house with 3 µg ml^−1^ nalidixic acid (NA). Honey samples submitted to EMAI were processed as described in Hornitzky and Clark [34]. Briefly, 75 ml of thin honey sample was combined with equal volume sterile PBS, mixed and centrifuged (3000 r.p.m. for 45 min). The majority of supernatant was removed, and 0.5 ml of supernatant and pellet was retained. The pellet was resuspended in the remaining liquid and heated at 80 °C for 15 min; then, 10 µl was streaked onto blood agar plates with NA and incubated for 4 days at 37 °C with 5% CO_2_. *P. larvae* colonies were confirmed by colony morphology, the catalase test [8,35] and MALDI-TOF MS using a Bruker Biotyper (Bruker Daltonik, Germany). A single *P. larvae* colony was then sub-cultured onto a blood agar plate with no NA and incubated at 37 °C with 5% CO_2_ for 2 days.

### DNA extractions

DNA extractions for short-read (Illumina) sequencing were performed using the QIAGEN DNeasy Blood and Tissue Kit (Cat # 69506), following the standard manufacturer’s protocol. Two small (1 µl) loopfuls of pure *P. larvae* cultures were resuspended in 180 µl of lysis buffer (20 mM Tris-HCl, 2 mM EDTA and 1.2% Triton) with 25 mg ml^−1^ lysozyme and digested overnight at 37 °C. After the addition of Proteinase K and Buffer AL, all samples were incubated at 56 °C for 30 min. Extracted DNA quality was checked on the NanoDrop 8 (ThermoFisher), and the samples retained if 260/280 and 260/230 ratios were between 1.8 and 2. DNA quantification was performed on the Qubit 4 (ThermoFisher) with the high specificity kit, and DNA extracts were stored at −20 °C for Illumina sequencing.

### Illumina sequencing and QC

Of the *P. larvae* isolates cultured, 371 with DNA extracts of sufficient quality and quantity were submitted to the University of Technology Sydney for WGS. Samples were prepared for sequencing using the Nextera system (Illumina, Cat # FC-131-1096) that included tagmentation of genomic DNA, amplification and sample balancing. DNA quantification was performed on the Agilent 2100 Bioanalyzer using the high-sensitivity DNA kit (Agilent, Cat # 5067–4626). After pooling, the samples were sequenced on the Illumina NovaSeq 6000 using the SP Reagent Kit v1.5 with 300 cycles (Illumina, Cat # 20028402).

### Public assemblies and sequencing reads

To compare the isolates sequenced in this study with previous studies, additional *P. larvae* reads were obtained from the Sequence Read Archive (SRA) at the National Center for Biotechnology Information (NCBI). Five hundred forty datasets were downloaded from the SRA in late 2021, deposited mostly from European studies [23,30, 36], and were obtained using sradownloader (https://github.com/s-andrews/sradownloader) in combination with sratoolkit (https://github.com/ncbi/sra-tools). A further 163 *P*. *larvae* genomes from a New Zealand study [1] were downloaded from the SRA in October 2024. Initially, all SRA reads were quality checked by FASTQC (https://www.bioinformatics.babraham.ac.uk/projects/fastqc/). For each read, Fastp [37,38] was used to remove adapters and 10 bp from the start and 5 bp from the end of each sequence. The final quality assessment of trimmed reads was performed by FASTQC and summarized by multiQC [39]. A reference genome assembly for each of the five ERIC types was downloaded from the NCBI RefSeq. Genome assemblies GCF_002951875.1, GCF_002951895.1, GCF_002951915.1, GCF_002951935.1, and GCF_011220525.1 were used to represent ERIC types I-V respectively.

### Assembly QC and *P. larvae* species confirmation

All *P. larvae* genomes were assembled from their read files using SPAdes v3.15.5 [40]. Default parameters were used excepting *k*-mer lengths, where only 31, 51 and 71 bp lengths were used. Quality metrics of all assemblies were checked using Quast v5.0.2 [41], and each assembly was assessed for contaminating DNA through Blobtools2 v3.0.0 [42]. Identification of each *P. larvae* assembly was performed *in silico* with Kraken2 v2.1.2 [43] and MLST v2.19 (https://github.com/tseemann/mlst). This publication made use of the PubMLST website (https://pubmlst.org/) as described in Jolley *et al.* [44]. Genome assemblies failed QC if they were outside of the expected genome size (~3.8–5.2 Gb) and had over 540 contigs or an N50 of 20 000 bp or lower. Ultimately, 368 EMAI obtained isolates, 476 genomes from the SRA genomes and 158 from Binney et al. [1] were bioinformatically confirmed as *P. larvae* by MLST and Average Nucleotide Identity (ANI) and passed all QC analyses. The 5 ERIC-type reference genomes from NCBI RefSeq were also included to make a total of 1007 *P*. *larvae* genomes for this study.

### MLST European subset

To provide a reference for diversity comparisons and to avoid potential biases caused by sequencing studies of localized outbreaks, a 129 subset of MLST *P. larvae* data was extracted from supplementary documents of Papić *et al.* [30]. AFB genomes were only selected if denoted as originating from the continent of Europe (data with location unknown were ignored).

### Phylogenetic analysis

Multilocus phylogenetic analysis was performed on 1007 *P*. *larvae* genomes using the up-to-date bacterial core gene 2 (UBCG2) v3.0 [45] that compared 81 highly conserved bacterial genes between genomes. Concatenated gene alignments generated by UBCG2 were trimmed with trimal v1.5.rev0 [46] using the automated strictplus trimming method. Phylogenetic trees were inferred using IQ-Tree [47]. More expansive genome-wide comparisons were then investigated using ERIC-I isolate sequences through cgSNP analysis via Snippy (https://github.com/tseemann/snippy) v4.6.0 using default settings and a *P. Larvae* ERIC-I reference assembly (RefSeq: GCF_002951875.1). After preliminary analyses, as well as referencing previous studies [48,49], we used a cgSNP relatedness threshold of cgSNPs ≤20 to identify isolates as belonging to the same outbreak cluster. The Fasta alignment file from Snippy was also converted into a cgSNP distance matrix using the snp-dists script (https://github.com/tseemann/snp-dists), and a heatmap was generated from the matrix using a custom R-script, Snp_heatmap.R (see Supplementary Methods). The SNP alignement was finally prepared for phylogenomic tree inferrence by removing non-standard nucleotide characters with snippy-clean_full_aln, recombination removal using gubbins v3.1.6 and core alignment generation using snp-sites v2.5.1. Phylogenetic trees were inferred with IQ-Tree v2.3.0 [47] using substitution model TVM+F+ASC+R3 and 1000 bootstrap replicates; the tree was rooted using minimum variance via the FastRoot.py script [50].

### Single beekeeper *P. larvae* genomic variation

We examined the genomic variation within an AFB-positive site owned by a single beekeeper. For this intra-apiary study, samples were collected from individual combs within beehives that were in close proximity and from which *P. larvae* isolates were obtained and subjected to WGS. These genomes were then analysed for genomic cgSNP differences using the cgSNP pairwise workflow described above.

## Results

### Isolate cohort observations and QC

**Fig. 1.**
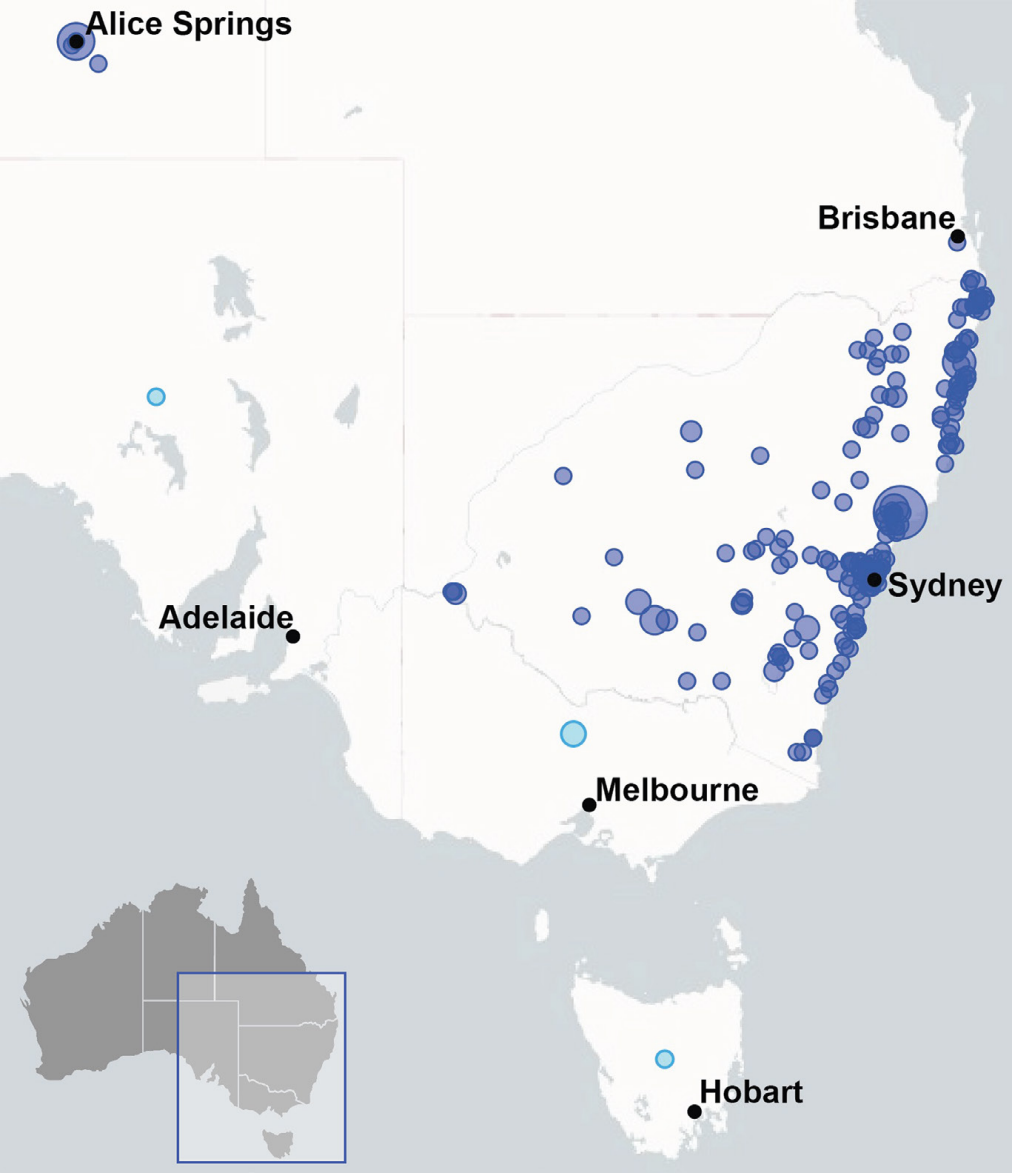
Map of AFB sampling sites. The 355 *P*. *larvae* Australian isolates (368 total minus 14 internationally sourced) examined in this study were obtained from all states and territories of Australia (except Western Australia), with the majority originating from SE Australia. Circles are proportional to the number of isolates obtained. In terms of location, the light blue points have been randomly placed near the centre of their Australian state, as no further geographical data were available.

A total of 368 isolate genomes sequenced in this study passed all sequence quality, species identification and sequence contamination assessments. More than 96% of these isolates originated from sites in all states and territories of Australia (except Western Australia) but were mostly from SE Australia (Fig. 1). Fourteen isolates were obtained from internationally sourced AFB infections. Of the SRA assemblies that passed all QC checks (*n*=472), the majority were from studies in Slovenia [30,36], Sweden [22,23] and New Zealand [1]. Reference genomes for all five ERIC types were also downloaded from the NCBI as detailed in the ‘Methods’ section. In total, 1007 *P*. *larvae* genomic assemblies were selected for further analysis. IDs for all 1007 genomes used are described in Table S1, and Illumina sequencing QC results for all 368 isolates sequenced in this study are outlined in Table S2. For simplicity, the isolates sequenced in this study (excluding those collected from international sources) will be referred to as Australian isolates. Some additional sequences sourced from Australia and New Zealand (but not sequenced in this study or Binney *et al.* [1]) were identified in public databases. One was obtained from Australia (ERR274208, ERIC-II) and three from New Zealand (ERIC-I: ERR274156 and ERR274157; ERIC-II: ERR274167). All 1007 genomes were identified as *P. larvae* by the MLST and Kraken2 applications, and all had ANIs of at least 99.6% or higher when compared against the genome of *P. larvae* DSM 7030^T^ and were close to the expected genome size (~4.3 Mb for ERIC-I) and had more than 540 contigs and an N50 of 20 000 or less.

Isolates collected as part of this study were distributed amongst the population, excluding the intra-apiary dataset below. Most isolates were sourced from separate individual beekeepers, with a small minority submitting multiple smears where isolates were obtained. The mean number of samples/beekeepers for this study is 1.85, with both the mode and median being 1.

### Australian isolates show decreased diversity compared with international isolates

To estimate the amount of genetic diversity present within Australian *P. larvae* isolates, we first examined and compared them to international isolates through *in silico* MLST. Europe and New Zealand (incl. Pacific Islands) are currently the only two international regions with significant numbers of *P. larvae* isolate genomes that can allow for diversity comparison. To better understand the relative diversity of Australian populations, we compared our dataset to existing genomic surveys of Europe and New Zealand [1,30]. We also examined genomes collected from the NCBI SRA, excluding Australian- and New Zealand-sourced genomes, to compare Australasian and international populations.

**Fig. 2.**
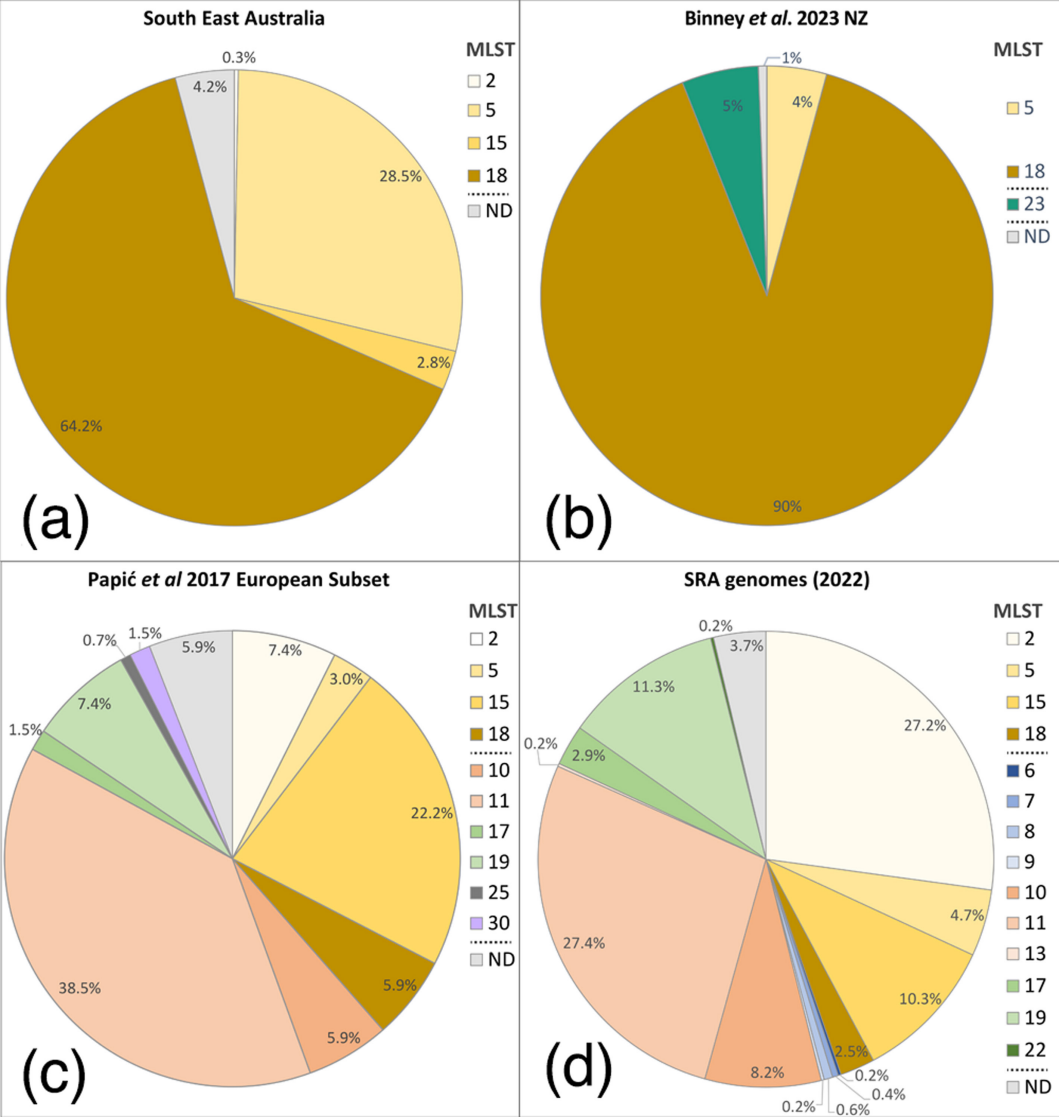
Pie graphs showing the diversity of worldwide *P. larvae* isolates genotyped with MLST. (**a)** Australian *P. larvae* isolates (*n*=355) from this study (excluding 14 non-Australian isolates). (**b)** New Zealand isolates (*n*=163) [1]. (**c)** European isolates (*n*=128) sourced from Papić *et al.* [30]. (**d)** SRA genomes from P. larvae isolates (*n*=476), excluding Australian and New Zealand sequences. Detected sequences are shown in figure legends with ND (no data) representing unidentified STs.

Australian and New Zealand *P. larvae* isolates showed similar MLST profiles and comparable population diversity (Fig. 2). ST18 was dominant in both countries, whilst ST5 was also found in both countries but more commonly in Australia. European populations have a higher diversity of MLST genotypes when compared with Australia and New Zealand, with 14 genotypes found in the European subset and only 5 found in Australia and New Zealand. Furthermore, sequence types (STs) that are prevalent in Australia and New Zealand (ST5 and ST18) are found in Europe and international datasets at lower abundance (<6%). In contrast, ST15 and ST2 represented a minor proportion of Australian isolates (2.8% and 0.3%, respectively) yet made up 10.3 and 27.2% of genomes from other countries and 22.2% and 7.4% from Europe. Interestingly, the frequency of ST18 and ST5 did not appear to change over time in Australian populations, whilst ST2 and ST15 were too rare for reliable time observations. Additionally, major MLST types (ST18 and ST5) were highly geographically dispersed, but minor types (ST2 and ST15) showed more localization, likely a consequence of their lower number of detections. Note that for 14 Australian *P. larvae* collected from an isolated region of central Australia (near Alice Springs, Fig. 1), ST5 was dominant (11 out of 13 isolates).

**Fig. 3.**
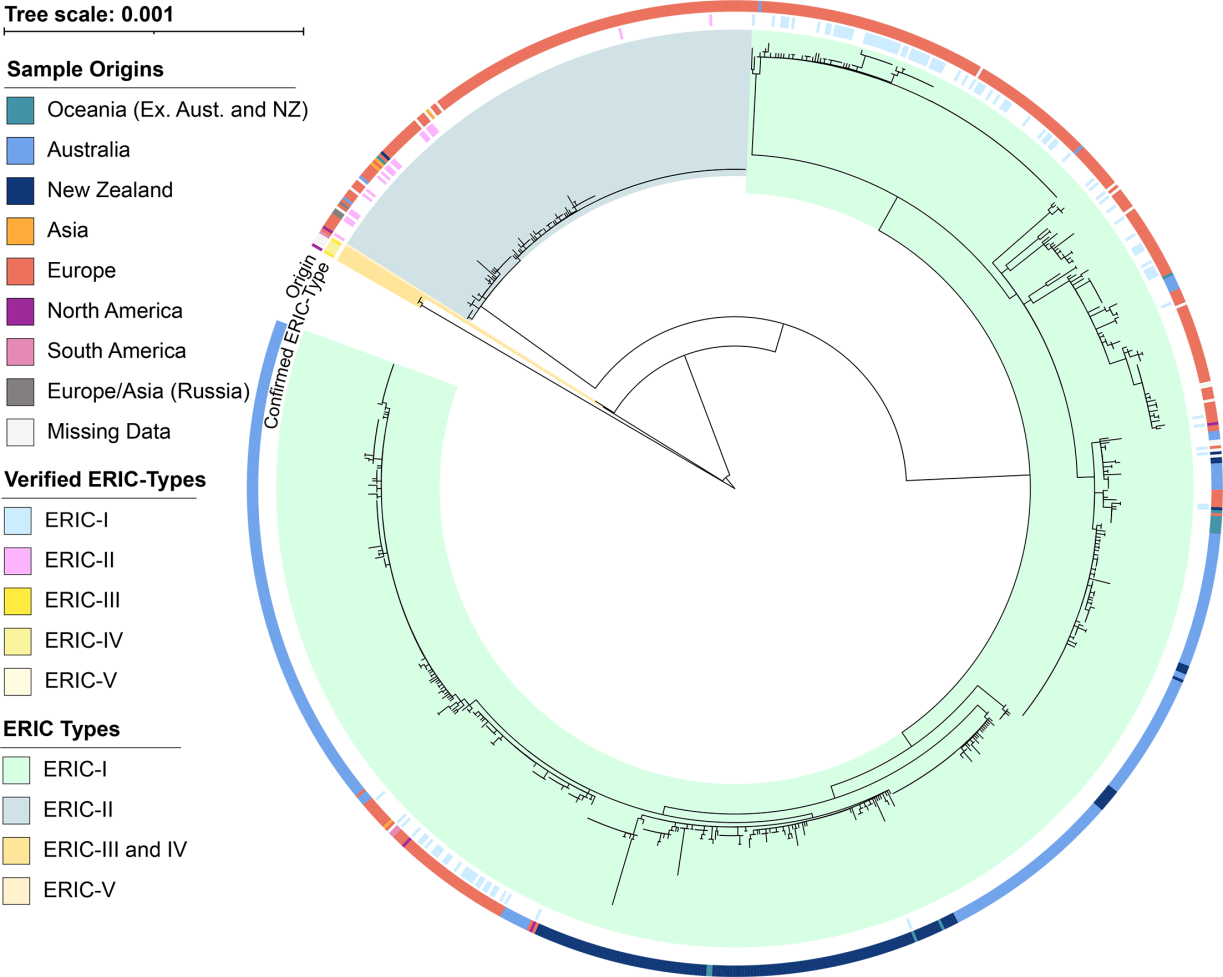
Multilocus phylogenetic tree generated by UBCG2 of 1007 P. larvae genomes. The branches are shaded to indicate likely ERIC types, with green for ERIC-I; blue-green for ERIC-2; and yellow for ERIC-III, -IV and -V. The inner colour wheel displays genomes with experimentally determined ERIC types. The outer colour wheel shows an isolated region of origin (if known).

To understand the evolutionary relationships between the 1007 *P*. *larvae* genomes, we employed UBCG2 to create a multilocus phylogenetic tree using 81 predefined loci [45] (Fig. 3). In the resulting tree, most isolates grouped into two discrete clades. One clade contained all confirmed ERIC-I reference genome, and several SRA genomes were experimentally identified as ERIC-I [23,30, 36]. The other large clade similarly consisted of the ERIC-II reference and a number of SRA genomes experimentally identified as ERIC-II [30]. These clades were classified as the ERIC-I and ERIC-II clades of *P. larvae* for this study. The remaining branches contain confirmed ERIC-III, ERIC-IV and ERIC-V isolates [3,24]. All remaining Australian isolates from this study were found within the ERIC-I clade, as were all isolates originating from New Zealand. Eleven of the 14 internationally sourced isolates sequenced in this study were found outside the clades or branches containing Australian and New Zealand isolates, although 1 from Fiji (T469) and 2 from New Caledonia (T71 and T72) were within a large clade of New Zealand-sourced genomes and closely related to several Australian isolates.

### AFB diversity is low in isolates collected from a single beekeeper

**Fig. 4.**
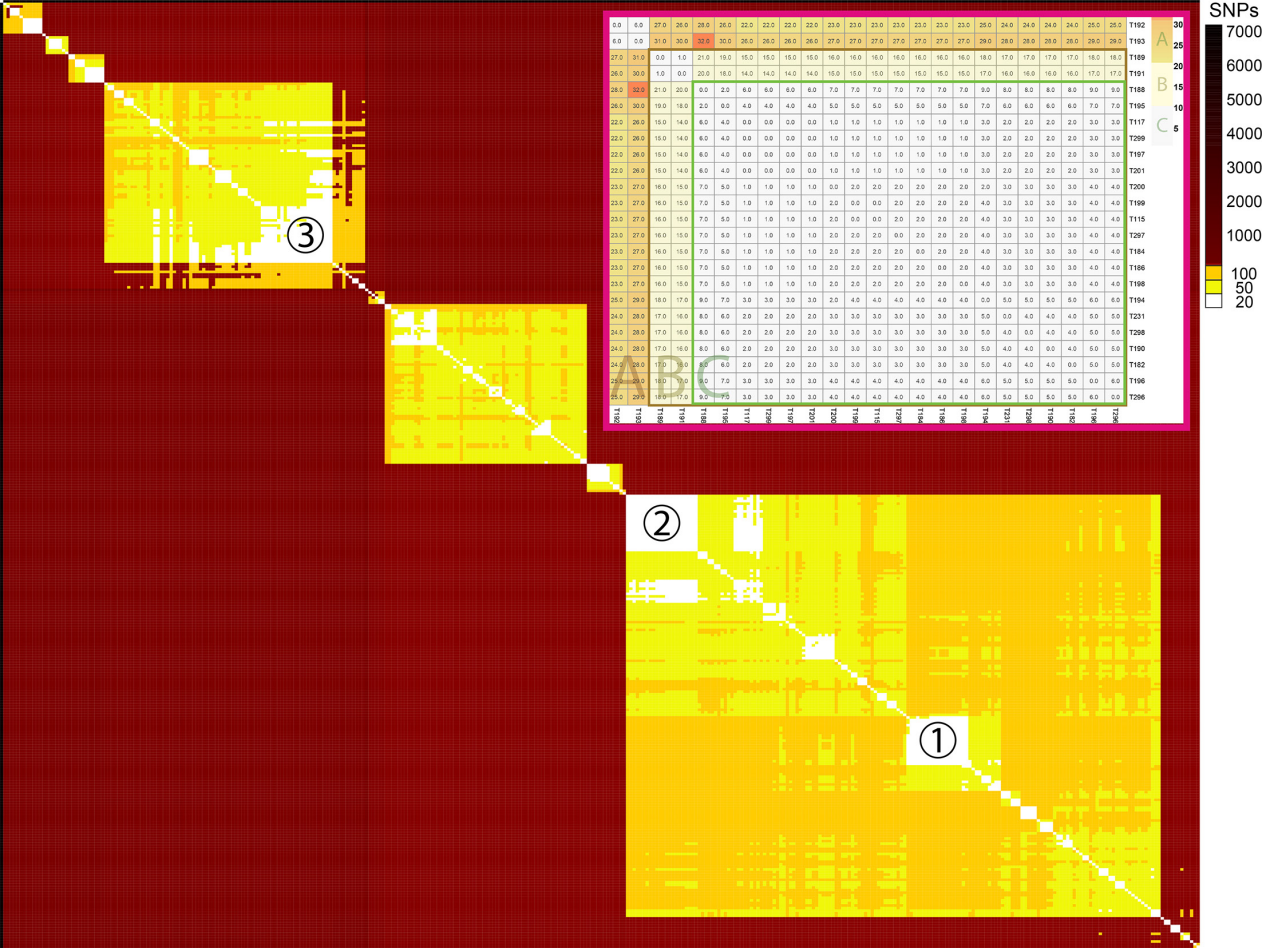
cgSNP pairwise comparison heatmap of 368 *P*. *larvae* genomes sequenced in this study against the ERIC-I reference sequence (RefSeq accession: GCF_002951875.1). Excluding the insert in the top right corner, the white cells represent small pairwise differences between genomes of 20 cgSNPs or less. Yellow is 21 to 50 cgSNPs and orange is a difference between 51 and 100 cgSNPs. Clusters-1 and -3 potentially represent an AFB outbreak from a single source. Cluster-2 isolates were all from a highly sampled site included to investigate intra-site genomic diversity from a putative single AFB origin. The top right insert represents all 24 isolates from an intra-apiary study (including 22 isolates from cluster- 2). Rectangle C represents a large core with cgSNP values ≤9, and rectangle B contains cgSNP values ≤21, whilst section A represents cgSNP values ≤32.

To identify clusters of high similarity that were potentially linked to AFB outbreaks, the 368 *P*. *larvae* isolates sequenced in this study were subjected to pairwise cgSNP comparisons between genomes using Snippy (Fig. 4). Several studies of AFB genomics have postulated that a genomic tracing scheme could be used to trace and control AFB detections [22,23, 30, 36]. To examine clusters of relatedness within samples collected from a single beekeeper, we sequenced 24 isolates from a single beekeeper with AFB-positive hives. In these isolates, we found diversity to be very low, with pairwise cgSNP distances less than or equal to 32 cgSNPs (Fig. 4, insert). Of these, a core of 20 from the 24 isolates had cgSNP distances ≤9 (Fig. 4, upper right insert, label C).

### Clusters of high similarity found in Australian-sourced AFB

Previous studies have indicated that 20 cgSNPs or less, ≥0.90 bootstrap support and monophyletic tree topology could represent an infection from a shared origin [48], so to this end, we further investigated 3 of the larger cgSNP clusters seen in Fig. 4. Genome cluster-1 comprised 18 isolates originating from multiple apiaries with a maximum cgSNP difference of 21. As expected, the intra-apiary isolates also formed a cluster (Fig. 4, cluster-2) of 22 isolates with a maximum cgSNP difference of 21 (Fig. 4, upper right insert, A and B) – also see above. Similarly, cluster-3 had very low cgSNP differences of 20 or less, which was particularly notable as 7 of the 14 were from historical samples obtained between the late 1980s to the mid-2000s.

### Resolving power of cgSNP

**Fig. 5.**
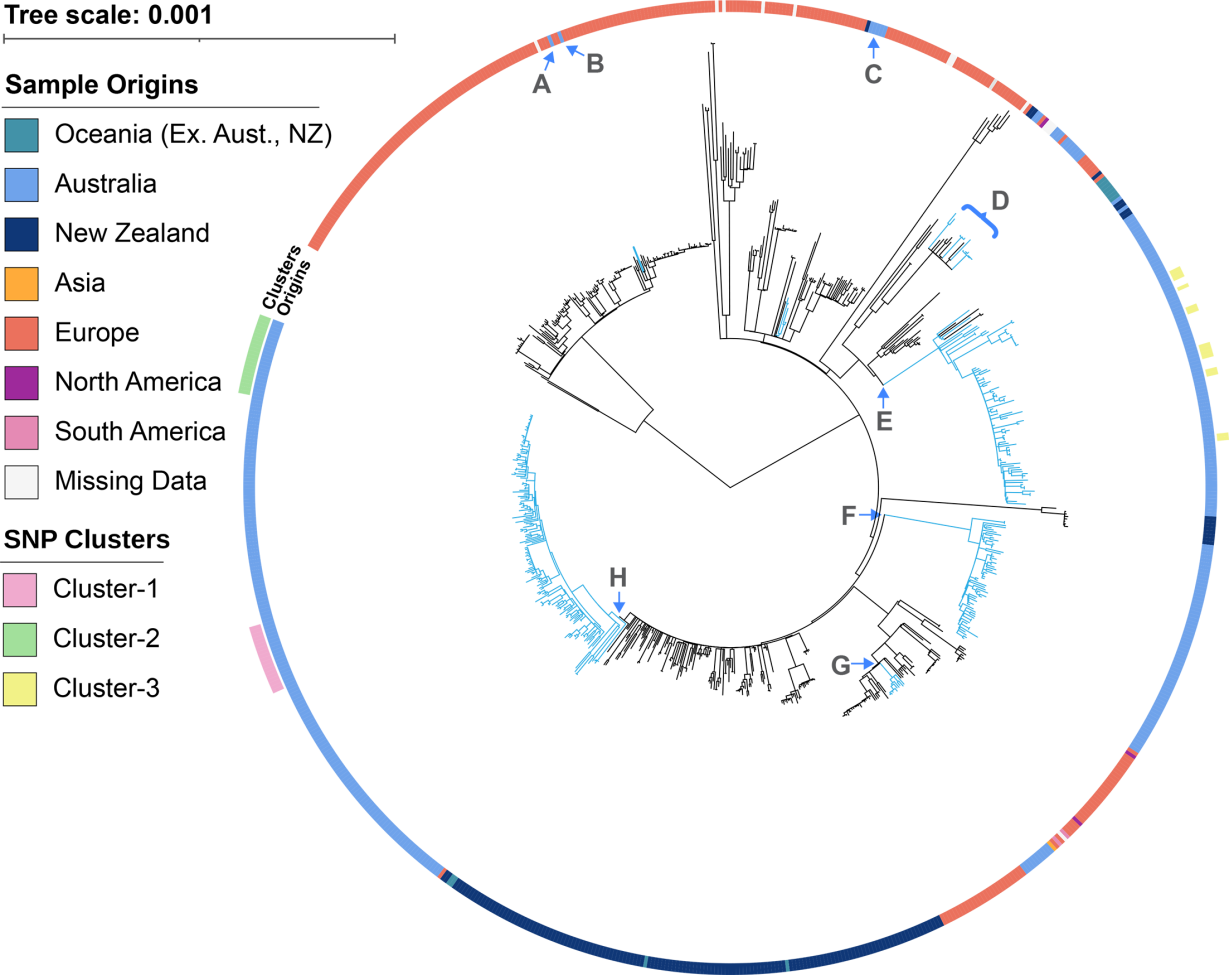
Phylogenetic tree of cgSNP alignments of 828 *P*. *larvae* ERIC-I genomes. The outer (discontinuous) ring shows the three clusters observed in Fig. 4. The inner ring shows the isolates originating from either Australia (blue), New Zealand (dark blue), Europe (red) or the other continents or regions which have fewer genomes available. The letters A, B, C, F, G and H represent clades of Australian *P. larvae* genomes, whilst D and E represent two clades of mixed origins that include Australia, New Zealand, Oceania (excluding Australia and New Zealand), Europe and North America.

The cgSNPs identified using Snippy were further analysed with maximum-likelihood phylogenetics (Fig. 5). In terms of the three isolate clusters from Fig. 4, the cgSNP phylogenomic tree of Fig. 5 mostly correlated with the cgSNP heatmap results of Fig. 4. We observed cluster-1 to be monophyletic (Fig. 5, pink highlight), with a bootstrap value of 0.879 and isolate origins dispersed over an approximate triangle of 450×500×350 km. Moreover, compliance officers from the NSW Department of Primary Industries (DPI) confirmed that these cluster-1 isolates originated from three commercial sources linked to one operator. Two of these operations had interconnected practices or transfer of material, with the third obtaining a queen bee from one of the other commercial operators. Cluster-2 consisted of 22 of the 24 intra-apiary isolates mentioned above with a bootstrap value of 0.96 and, as expected, was also monophyletic (Fig. 5, lime-green highlight). Cluster-3, similar to cluster-1, encompassed another large triangular area (700×550×400 km) and had cgSNP differences of 21 or less, with a high bootstrap value of 0.965. However, cluster-3 isolates were not monophyletic with isolates distributed within a large clade of 69 Australian isolates, and indeed, 7 of the 14 cluster-3 isolates (Fig. 5, yellow highlight) were from historical samples obtained between the late 1980s until the mid-2000s.

### cgSNP analysis shows eight Australian AFB lineages affecting honeybees

Using the high resolving power of cgSNP phylogeny, Australian-sourced isolates consisted of 3 major clades (Fig. 5, E, F and H), 2 smaller clades (Fig. 5, C and G), another 3 smaller clades and 1 leaf within a mixed clade of 18 genomes (Fig. 5, D) that included 2 European and 1 North American genomes and 2 single isolates (Fig. 5, A and B). Interestingly, genomes A and B (Fig. 5) both originated from Australia (A from our study and B from the SRA: ERR4854272), yet both were within a large European clade (Europe, 129; Australia, 2; and unknown, 1). In general, there was little mixing of the Australian, New Zealand and European types with some exceptions. In Fig. 5, letters A, B, C and G represent one or more Australian genomes within a clade of European genomes. Similarly, individual or small numbers of Australian genomes are within clade-D as well as several genomes from Oceania (excluding Australia), Europe and North America (one genome).

### Australian AFB cgSNP-type distribution

Although the number of isolates sequenced in this study outside of SE Australia was low, our results suggest a spread of *P. larvae* strains throughout Australia. One group of ten highly related isolates was sourced from four Australian states (Fig. 5, clade E, closest to parent node), whilst some Central Australian isolates were closely related to SE Australian strains (Fig. 5, clade G). Additionally, three isolates sourced from the Northern regions of Australia were also highly related to SE Australian isolates (Fig. 5, clade H).

## Discussion

The ability to genotype an infective agent is a significant tool in any effort to track and control the spread through a host population. This is of particular importance for *P. larvae* – the cause of AFB in European honeybees, as the only effective control of this disease is colony destruction. An oral vaccine has recently been developed against *P. larvae* [11]. However, even if proven effective, the vaccine is unlikely to fully eradicate AFB given its spore-forming nature and ability to hide within wild honeybee populations. There are also gaps in knowledge of the *P. larvae* population within Australia, which should be addressed before any vaccine trials are attempted within Australia. Using a total of 1007 genomes, we examined the diversity of Australian populations in comparison with other continents or regions and investigated whether cgSNP genotyping has sufficient discriminatory power to effectively trace a *P. larvae* outbreak within Australia to a single source. Although this has been demonstrated for *P. larvae* in other countries [23,30], it has not been demonstrated within the Australian population, which our results indicate has a lower genetic diversity than European populations, where most of the available *P. larvae* sequences are currently sourced from. As found in other countries, the ability to trace AFB in Australia to the strain level would be a powerful asset, giving compliance officers an additional tool to quickly detect the source of an outbreak and restrict its severity.

The European honeybee (*A. mellifera*) is believed to have been successfully introduced initially into Australia in 1822 (Sydney Gazette, 12 April 1822, page 2, see https://trove.nla.gov.au/ newspaper/article/2180928). It is likely that *P. larvae* strains were additionally introduced during the early British colonization of Australia. However, several modern sources of introduction exist, including AFB spores found in imported honey, stowaway hives transported by global shipping or illicit trade of queen bees. Of the five *P. larvae* ERIC types, only ERIC-I, the most pathogenic in terms of hive destruction, was identified within Australian isolates sequenced in this study. Similarly, there were only four Australian *P. larvae* MLST types identified. In contrast, there were 14 MLST types identified within publicly accessible *P. larvae* genomes and 10 within the strict European context we examined from Papić *et al.* [30], with dominant Australian types forming a low percentage of European populations. We also observed that Australian (and New Zealand) isolates typically formed discrete clusters in multilocus and cgSNP phylogenetic analysis amongst a background of European isolates, potentially indicating a low number of introductions. These results suggest that ancestors of *P. larvae* ST18 and ST5 isolates may represent early founders and/or have an evolutionary advantage that helped them persist within the generally warmer and drier Australian environment. However, the relatively few mixed origin phylogenetic clades observed in this study (i.e. clades containing isolates from multiple continents) imply that, despite numerous possible routes of transmission, successful importation and establishment of AFB into Australian hives from international sources is rare. Further sequencing of *P. larvae* from other continents, especially North America and Asia, would improve the understanding of international AFB transmission.

Our observations indicate that Australian *P. larvae* strains currently have a lower genetic diversity than populations from international sources. We also examined how modern strains compare with historical Australian isolates collected over a 30–40-year period. We identified 8 cgSNP clusters in our full dataset of 368 isolates. Three of the 8 Australian clades (Fig. 5, C1, E and F) identified in cgSNP phylogenetics did not contain historical isolates (collected prior to 2010), and 2 of these clades consisted of less than 15 genomes. Previous studies of *P. larvae* diversity in Australia have used lower sample numbers, but the results appear to be consistent. Ten unique PFGE types were identified from 33 isolates sourced from Eastern and Central Australian states [51]. Although PFGE results cannot be directly compared to cgSNP genotype clusters, this previous study and sequencing of historical isolates performed here does indicate that *P. larvae* diversity in Australia has not greatly increased in the last 30–40 years.

Intra-apiary diversity has been previously examined in genomic surveys of *P. larvae*. Analysis of outbreak clusters during validation of the *P. larvae* cgMLST scheme revealed isolates from multiple separate clusters in samples provided by individual beekeepers [23]. Additionally, previous work has attempted to identify diversity within defined AFB outbreaks [52]. In contrast, we identified less diversity in the single apiary examined in this study, with a maximum cgSNP distance of 32 found in 24 genomes sourced from the same apiary and all found to be from the same 7-gene MLST type. We did identify small but highly related clusters in these sequences, with the primary cluster consisting of 20 isolates and <10 cgSNPs but also two slightly distant groups consisting of two isolates each (Fig. 4, insert). Further sequencing studies involving multiple isolates from single apiaries could determine if this diversity is common and, if so, could potentially be used to recommend a higher numerical threshold to identify *P. larvae* outbreak clusters. Genomic sequencing surveys and epidemiological tracing should continue to consider sequencing multiple isolates from individual apiaries to maximize understanding of *P. larvae* diversity and AFB transmission.

To better discriminate Australian *P. larvae* isolates across the SE Australian population, we performed a cgSNP analysis on 368 isolates, finding several highly related clusters of isolates (Fig. 4). We adapted a previously determined cut-off value of cluster threshold (CT) ≤20 cgSNPs as one important parameter to help define a single strain of Australian *P. larvae* isolates [48]. Considering previously observed diversity in international inter-aviary datasets, this was chosen as a conservative threshold incorporating previously published guidance on cgSNP comparison and results from our intra-apiary study [48]. From this, we focussed on three of the larger clusters observed with a CT ≤20 cgSNPs. Cluster-1, with a maximum cgSNP difference of 21 (in only 1 genome pair), consisted of 18 isolates from 2 apiarists that had origins encompassing a roughly triangular area spanning hundreds of kilometres on all sides. The potential for cluster-1 to represent an infection from a single source was later confirmed by bee disease tracing officers from the NSW DPI, who found that this AFB outbreak had spread via a contaminated queen bee transferred from one apiarist to another. As expected, given that the 22 cluster-2 isolates were part of an intra-apiary study at an isolated site belonging to a single apiarist, all cluster-2 isolates were found to be from a single strain. Notably, an additional two isolates from the same intra-apiary study did not cluster nearby and had slightly higher cgSNP values ranging from 22 to 32. This may indicate that the cgSNP cut-off used was too low or that perhaps a new strain, strain evolution, or molecular clock rate variability or sample collection times [53] were the cause. For the cluster-3 isolates, like cluster-1, the 14 isolates were also found to originate from an area that spanned hundreds of kilometres. However, for this set of isolates, the idea of a single origin of infection was highly unlikely, due to 7 of the 14 originating from historical samples spanning the late 1980s to the mid-2000s. The latter results reinforce the suggestion that numerical cgSNP thresholds can be misleading and that a number of parameters, such as monophyly and inclusion of closely related background sequences, are needed to identify a single outbreak cluster [48].

To determine if background sequences and phylogenetics could improve cluster detection, we constructed a cgSNP tree (Fig. 5) that incorporated 1007 Australian and international *P. larvae* genomes. The monophyletic nature of cluster-1 and high bootstrap value (0.879) and cgSNP distance ≤20 strongly suggest that this cluster represents a single outbreak strain. Despite the observed bootstrap value being below the suggested cut-off (0.9), it is close, and small variations are expected [49]. For the cluster-2 isolates, as expected from the intra-apiary study, all had parameters identifying them as belonging to a single strain (cgSNP ≤20 values, monophyletic, with a bootstrap of 0.96). In contrast, whilst the cluster-3 isolates also had low cgSNP distance, the cluster was observed to be polyphyletic with all 14 isolates distributed within a large clade of 69 Australian isolates. Thus, using the basic parameters and values suggested by Pightling *et al.* [48] and informed by our results, we were able to identify unique strains of *P. larvae* within the Australian context. To date, whilst cgSNP analysis has already been successfully used to investigate and identify unique strains from several different species [54,56], including *P. larvae* [30], this is the first time this has been shown for Australian *P. larvae* isolates, which display a low population diversity.

Despite the apparent reduced genetic diversity of the Australian *P. larvae* isolates in comparison with those from Europe, AFB has clearly survived and spread throughout Australia. Here, we observed that abundant *P. larvae* MLST types were geographically dispersed, which is likely due to the migratory nature of commercial beekeeping operations in Australia [30,57]. Considering this dispersal, it is interesting that we did not detect any ERIC-II isolates in this study, despite previous examples of Australian-sourced ERIC-II reported [30]. ERIC-II was similarly not detected in a survey of New Zealand isolates despite previous reports from the same study [30]. ERIC-I and ERIC-II are considered slow and fast killers of bee larvae, respectively [25]. However, the opposite is true at the colony level where ERIC-I is considered more devastating to bee colonies, potentially due to the behaviour of nurse bees in response to fast-killing *P. larvae* strains [25]. Consequently, the reduced severity of ERIC-II infections at the colony level may lead to under-detection of ERIC-II in surveys that rely on samples from routine inspections or diagnostic submissions. However, ERIC-II strains are often observed to be more prevalent than ERIC-I strains in these types of studies of European populations [36,58]. Additionally, we included *P. larvae* isolated directly from honey samples in this survey and detected no evidence of *P. larvae* ERIC-II. The low prevalence of ERIC-II in Australian and New Zealand *P. larvae* could potentially be due to the isolated nature of Australian and New Zealand *P. larvae* under national quarantine regulations but could also be due to a selective advantage for ERIC-I in different climates.

## Conclusion

Our results indicate that *P. larvae* is less genetically diverse within Australia compared with European populations but highly similar to *P. larvae* in New Zealand. The majority of the commercial beekeeping industry occurs within the area of SE Australia, from which many Australian samples in this study have originated. We found a low level of genomic diversity after ERIC and MLST, as well as Multilocus sequence analysis (using UBCG2 with its subset of highly conserved core genes) and cgSNP analysis. Despite the reduced genomic diversity, we found that genotyping through cgSNP analyses was able to discriminate between, and track, different strains of *P. larvae* within Australia. In at least one case, cgSNP analysis was confirmed by epidemiological tracing and allowed independent identification of an AFB outbreak to a single source. Nonetheless, cgSNP-based analysis requires closely related, high-quality, near-complete reference sequences and publicly available data to assist inter-study compatibility. A more flexible alternative such as cgMLST or multilevel genome typing is a potential alternative.

## Supplementary material

10.1099/mgen.0.001374 Uncited Supplementary Material 1.

10.1099/mgen.0.001374 Uncited Supplementary Material 2.

## Abbreviations

AFB: American foulbrood
cgMLST: core-genome MLST
cgSNPs: core-genome SNPs
CT: cluster threshold
DPI: Department of Primary Industries
EMAI: Elizabeth Macarthur Agricultural Institute
ERIC: enterobacterial repetitive intergenic consensus
MLST: multilocus sequence typing
NA: nalidixic acid
NCBI: National Center for Biotechnology Information
QC: quality control
SE: south-eastern
SRA: Sequence Read Archive
ST: sequence type
UBCG2: up-to-date bacterial core gene 2
WGS: whole-genome sequencing
