# Supplementary material for: Genomic diversity and tracing of Paenibacillus larvae in Australia: implications for American foulbrood outbreak surveillance in low-diversity populations
Source: Microb Genom. 2025 May 6;11(5):001374. doi: 10.1099/mgen.0.001374 (PMC12163730; doi:10.1099/mgen.0.001374)
Supplement: Uncited Supplementary Material 2. [file mgen-11-01374-s002.pdf]

Contents

Scripts 1
Snp_heatmap.R 1
newickTreeAndHeatmap.R 3

Supplementary file associated with the article - Genomic Diversity and Tracing of *Paenibacillus larvae* in Australia: Implications for American Foulbrood Outbreak Surveillance in Low Diversity Populations.

Scripts

Snp_heatmap.R. Creates a heatmap of SNP pairwise comparisons from each P. larvae isolate

```
library(pheatmap)
library(dplyr)
library(RColorBrewer)
library(gridExtra)
library(grid)
#install.packages("viridis")
library(viridis)

#################### SAVE HEATMAP Function ####################
#Save pheatmap function
save_pheatmap <- function(x, filename, width=1500, height=800) {
  stopifnot(!missing(x))
  stopifnot(!missing(filename))
  png(filename = filename, width = width, height=height)
  grid::grid.newpage()
  grid::grid.draw(x$gtable)
  dev.off()
}
#################### END FUNCTION ####################

setwd("path/to/input/tab-separated-table")
dataTable = read.delim("input_filename.txt",
           stringsAsFactors = FALSE)
row.names(dataTable) = dataTable[,1]
dataTable[,1] = NULL

dataTable = dataTable[-1, -1]

#Set the palette length for the colour ramp
paletteLength = 90
#Set the colour ramp to use in the heatmap
myColor = colorRampPalette(c("red", "black"))(paletteLength)
myBreaks = round(c(seq(0, 20, length.out = 20), seq(21, 50, length.out = 30),
                    seq(51, max(dataTable), length.out = 90)))
```

```
# # ALTERNATIVE
# myColor = colorRampPalette(c("darkred", "black"))(paletteLength)
# myColor = c("white", "#FFCC00", myColor)
# myBreaks = c(1, 21, 51, seq(100, max(dataTable)/2, length.out = 30), seq(150,
max(dataTable), length.out = 100))


    # # _____>Plot the heatmap in basic colours<_____
    # sigProteins_heatmap = pheatmap(dataTable, color = colorRampPalette(c("khaki",
"coral"))(140),
    #                                cluster_rows = TRUE, cluster_cols = TRUE,
show_rownames=TRUE, display_numbers = FALSE,
    #                                width = 10, height = 10, fontsize = 1, breaks
= myBreaks,
    #                                annotation_names_col = FALSE)
    #
    # # _____>Plot the heatmap in basic colours<_____
    # sigProteins_heatmap = pheatmap(dataTable, color =
colorRampPalette(c("lawngreen", "black", "firebrick1"))(140),
    #                                cluster_rows = TRUE, cluster_cols = TRUE,
show_rownames=TRUE,
    #                                display_numbers = TRUE, width = 10, height =
10, fontsize = 1,
    #                                breaks = myBreaks, annotation_names_col =
FALSE,
    #                                clustering_distance_cols = "manhattan" ,
clustering_distance_rows = "manhattan",
    #                                clustering_method = "complete")


# Plot the heatmap in softer colours (better differention)
sigProteins_heatmap = pheatmap(dataTable, color = myColor, cluster_rows = TRUE,
                               show_rownames=TRUE, breaks = myBreaks, width = 10,
                               height = 10, fontsize = 0.8,
                               #display_numbers = TRUE, number_color = "white",
                               annotation_names_col = FALSE,
                               clustering_distance_cols = "correlation",
                               clustering_distance_rows = "correlation",
                               clustering_method = "complete")

# sigProteins_heatmap = pheatmap(dataTable, color = viridis(140, option = "E"),
#                                 cluster_rows = TRUE, display_numbers = FALSE,
#                                 show_rownames=TRUE, width = 10,
#                                 height = 10, fontsize = 1,
#                                 annotation_names_col = FALSE,
#                                 clustering_distance_cols = "manhattan" ,
#                                 clustering_method = "complete")


fillerRectangle = grid.rect(width = 0.5, height = 0.5, gp = gpar(fill = "white",
col = "white", alpha = 0.8))
grid.arrange(fillerRectangle, sigProteins_heatmap[[4]], fillerRectangle, nrow=1,
widths = c(1,20,1))

# Save the grobs (including the pheatmap heatmap) to disk
png("pheatmap_pyani_correlation.png", width = 11.69, height = 8.27, res = 600,
units = "in") # Open a new pdf file
grid.arrange(fillerRectangle, sigProteins_heatmap[[4]], fillerRectangle, nrow=1,
widths = c(1,20,1))
dev.off()

pdf("pdf_filename_out.pdf", width = 11.69, height = 8.27) # Open a new pdf file
grid.arrange(fillerRectangle, sigProteins_heatmap[[4]], fillerRectangle, nrow=1,
widths = c(1,20,1))
dev.off()
```

newickTreeAndHeatmap.R. Combines a newick format phylogenomic tree with a heatmap. Merging based on sample names from the heatmap and tip names from the phylogenomic tree.

```
library(ape)
library(phytools)


# ------------------------------ User Input ------------------------------
pathToNewickFile = "path/to/newick/file"
pathToGenePresenceAbsence = "path/to/gene/presence/absence/file.Rtab"
outputFileName = "treeAndHeatmap"
treephy = phytools::read.newick(pathToNewickFile)
# -------------------------------------------------------------------------

setwd("path/to/working/directory")

genotypeNew <- read.table(pathToGenePresenceAbsence, sep="\t", header = TRUE,
stringsAsFactor=FALSE, check.names = FALSE)
genotypeNew_matrix_t <- t(genotypeNew)
colnames(genotypeNew_matrix_t) <- genotypeNew_matrix_t[1,]
genotypeNew_matrix_t <- genotypeNew_matrix_t[-1,]

genotypeNew_rowNames = row.names(genotypeNew_matrix_t)
genotypeNew_matrix_t <- apply(genotypeNew_matrix_t, 2 ,as.numeric)
row.names(genotypeNew_matrix_t) = genotypeNew_rowNames
genotypeNew_matrix_t = genotypeNew_matrix_t[, c(ncol(genotypeNew_matrix_t),
1:ncol(genotypeNew_matrix_t))]
# bob = genotypeNew_matrix_t[1:50, 1:100]

colors<-colorRampPalette(colors=c("white","dodgerblue4"))(20)
phylo.heatmap(treephy, genotypeNew_matrix_t, fsize = 0.26, length = 10,
split=c(0.5,0.5), labels=FALSE, colors = colors, legend=FALSE)

# PDF Output
sizeValue = 0.3
pdf(file = paste0("treeHeatmap.pdf"), width = 11.75, height = 8.25)
phylo.heatmap(treephy, genotypeNew_matrix_t, fsize = sizeValue, length = 10,
split=c(0.5,0.5), labels=FALSE, colors = colors, legend=FALSE)
dev.off()

# PNG Output
png(file="treeHeatmap.png", width=2400, height=1700)
phylo.heatmap(treephy, genotypeNew_matrix_t, fsize = sizeValue*4, length = 10,
split=c(0.5,0.5), labels=FALSE, colors = colors, legend=FALSE)
dev.off()
```
